# Editorial

**Published:** 1838-11-21

**Authors:** 


THE MEDICAL EXAMINER.

PHILADELPHIA, NOV. 21, 1838.

Our readers will perceive that we continue our reports of the Clinical Lectures delivered at the hospitals of this city during the winter session. The number of students is this year unusually large at both these institutions; and from the increasing interest which they manifest in the study of demonstrative medicine, we may confidently hope, that within a short period it will be regarded as an essential department of medical instruction.

The difficulties which attend this branch of medical instruction are still considerable, but are not insurmountable. They will in a great measure disappear, when its importance is fully appreciated both by the medical profession and the public at large. Clinical instruction cannot be rendered much more complete, until the students of medicine are convinced that a practical familiarity with disease should be attained previously to their entrance upon practice. An attempt to extend the present course of instruction would fail without their active co-operation, for no institution in this country is sufficiently powerful to exact from its pupils a larger sacrifice of time and labour than they are willing to yield. As soon as the students themselves are convinced of the necessity of increased facilities for instruction in any department of medicine, we have no doubt that they will be furnished to them; and although these facilities are of more difficult attainment, as regards clinical medicine, than most other branches of instruction, they may be overcome with patient perseverance.

